# Black phosphorous-based human-machine communication interface

**DOI:** 10.1038/s41467-022-34482-4

**Published:** 2023-01-03

**Authors:** Jayraj V. Vaghasiya, Carmen C. Mayorga-Martinez, Jan Vyskočil, Martin Pumera

**Affiliations:** 1 Center for Advanced Functional Nanorobots, Department of Inorganic Chemistry, Faculty of Chemical Technology, University of Chemistry and Technology Prague, Technická 5, 166 28 Prague, Czech Republic; 2 Department of Chemical and Biomolecular Engineering, Yonsei University, 50 Yonsei-ro, Seodaemun-Gu, Seoul, 03722 Korea; 3 Faculty of Electrical Engineering and Computer Science, VSB—Technical University of Ostrava, 17. listopadu 2172/15, 70800 Ostrava, Czech Republic; 4 Department of Medical Research, China Medical University Hospital, China Medical University, No. 91 Hsueh-Shih Road, Taichung, 40402 Taiwan

**Keywords:** Sensors and biosensors, Two-dimensional materials

## Abstract

Assistive technology involving auditory feedback is generally utilized by those who are visually impaired or have speech and language difficulties. Therefore, here we concentrate on an auditory human-machine interface that uses audio as a platform for conveying information between visually or speech-disabled users and society. We develop a piezoresistive tactile sensor based on a black phosphorous and polyaniline (BP@PANI) composite by the facile chemical oxidative polymerization of aniline on cotton fabric. Taking advantage of BP’s puckered honeycomb lattice structure and superior electrical properties as well as the vast wavy fabric surface, this BP@PANI-based tactile sensor exhibits excellent sensitivity, low-pressure sensitivity, reasonable response time, and good cycle stability. For a real-world application, a prototype device employs six BP@PANI tactile sensors that correspond to braille characters and can convert pressed text into audio on reading or typing to assist visually or speech-disabled persons. Overall, this research offers promising insight into the material candidates and strategies for the development of auditory feedback devices based on layered and 2D materials for human-machine interfaces.

## Abstract (web summary)

Tactile sensors in human-machine interaction systems can provide precise input signals and the necessary feedback between humans and machines. Here, the authors developed a black phosphorous-based tactile sensor array system that can provide touch into audio feedback.

## Introduction

With the rapid advancement of wearable electronic technology, tactile sensors have recently gained a lot of attention as critical components in touch displays, smart medical care, motion detection, electronic skin, and human-machine interface (HMI)^1–3^. A tactile sensor is a kind of sensor that detects changes to an electric signal by an applied force. Several types of tactile sensors have been developed to accurately sense and measure applied force based on piezoelectric^4,5^, piezocapacitive^6,7^, and piezoresistive^8–10^ processes. Among them, sensors that operate on piezoresistivity have attracted interest due to their simple device fabrication and low power consumption.

Active materials are the most crucial components in the construction of piezoresistive tactile sensors and must meet certain requirements including skin conformity, tunable metallic/semiconducting properties, and high porosity. In the past, carbon-based materials (*i.e*., carbon nanotubes and graphene), metal wire, polymers, and their composites have been used as active layers in sensors. Unfortunately, this method still suffers from high production cost and low yield^11,12^. Therefore, a new sensing active material is required to develop a highly sensitive piezoresistive tactile sensor. Two-dimensional transition metal carbides (*e.g*., Ti_3_C_2_ MXene) are potential choices for developing piezoresistive tactile sensor electrodes due to their adjustable interlayer spacing, simple synthesis process, high specific area, and excellent metallic conductivity^13–17^. MXene has previously been utilized to build a highly sensitive piezoresistive sensor^8,9,18,19^. To date, MXene-based tactile sensors have limited stability, which must need to be enhanced in the future^20^.

However, the black phosphorous- (BP) based sensor has received very little attention. BP possesses a variety of advantages over conventional active materials for piezoelectric or resistive sensors as previously stated^21–23^. BP comprises a puckered honeycomb lattice structure and has good carrier mobility, making it very sensitive and mechanically robust^24–26^. Unfortunately, the majority of BP-based sensors demonstrated in the structural analysis (*e.g*., bandgap modulation), research has been very limited to real applications such as human physiological signals monitoring and human-machine interfaces.

There is recent interest in HMI techniques that use touch sensing to establish real-time synchronized communication between humans and machines^27–29^. Although HMI needs to perform touch sensing with auditory feedback, the major emphasis has been on touch sensing and research on various types of sensing feedback is still limited. For instance, a Ti_3_C_2_-based tactile sensor was employed to perform real-time braille recognition^30^. However, recognized braille letter appears on the screen, which makes it harder to communicate with other vision-impaired persons. Wang et al. developed a refreshable braille display based on dielectric elastomer that can provide greater touch sensations in real-time^31^. But the device suffers from a lack of stability, low cost and simplicity of fabrication. Other kinds of braille display devices have also been developed by other scientists^32–34^. But they are strongly dependent on a high-voltage source, which may be harmful to visually impaired persons. Furthermore, existing tactile sensors have good sensing capability in a single device, when integrating multi-tactile sensors in one module many challenges exist such as signal crosstalk among the pixels and the need high power. Therefore, developing highly stable and robust a touch to audio braille recognition device for blind people is critical.

Here we reported a facile and cost-effective piezoresistive tactile sensor using BP@polyaniline (PANI) composite. With the superior carrier mobility of BP@PANI composite and the plentiful wavy fabric surface, the sensor can sense a wide range of pressure and exhibits reasonable response as well as cycle stability. Our solution offers low cost and low voltage operation while preserving reasonable sensitivity, linearity, durability and ease of integration into a wearable large-scale array. We develop an assistive auditory feedback device for visually disabled people, a prototype device using six BP@PANI-based tactile sensors that correspond to braille characters. This device can convert touch in various braille patterns into audio that will speak the alphabet. Best our understanding, the BP in tactile sensor and auditory feedback system have never been reported before. The proposed device is advantageous not only for blind people but also for people with speech and language difficulties.

## Results

### Fabrication, characterization, and sensing mechanism

**Fig. 1:**
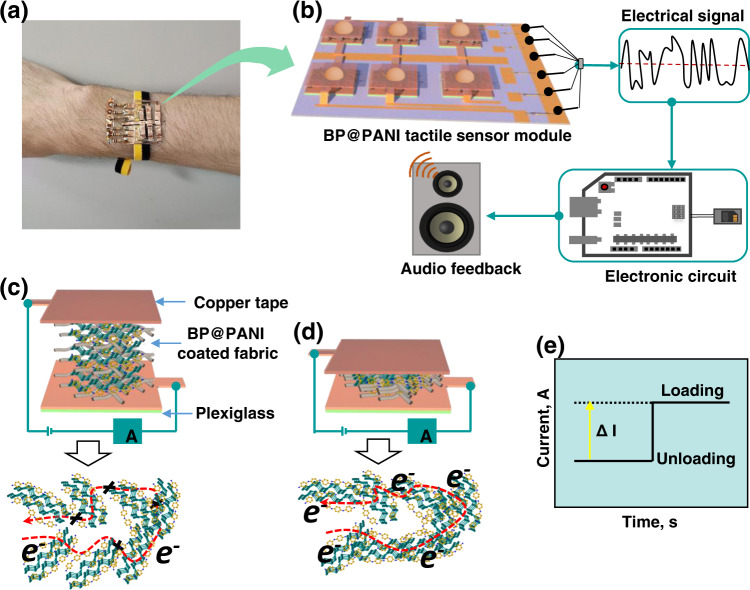
Conceptual diagram of the BP@PANI-based tactile sensor for an auditory human-machine interface. **a** Wearable auditory system attached on the hand of vision challenged person. **b** Six-pixel tactile sensor array that meets the typical braille character set to read alphabet letters corresponding to pressed sensor patterns. Schematic illustration and sensing mechanism of BP@PANI-based tactile sensor under unloading (**c**), loading (**d**), and corresponding current response (**e**).

The wearable tactile sensors with piezoresistive properties are highly desirable for HMI. In this paper, we develop a piezoresistive tactile sensor based on BP@PANI composite that provides good wearability, stability, and sensitivity over a wide pressure range. The fabrication process of the BP@PANI-based tactile sensor is depicted in Supplementary Fig. 1 (more information is provided in the Experimental Section). Supplementary Figure 2 shows the structural geometry of BP@PANI-based tactile sensor, where 1.85 mm in thickness, 0.73 g in weight, 1.5 cm in width, and 1.5 cm in height. The benefit of in situ polymerizations of PANI is that it can form a multilevel nano-rough texture on the surface of BP, which can enhance the mechanical sensing ability of the tactile sensor. Taking advantage of the tactile sensor based on BP@PANI with great sensitivity over a wide range, the sensor is used to convert touch into audio feedback (Fig. 1a). To showcase this application in the HMI field, we created a prototype wearable tactile sensor matrix that comprises a braille character set and allows the user to hear auditory feedback in response to the braille text they are pressing (Fig. 1b). This concept provides braille display portability and makes it more durable and comfortable to wear.

To better understand the sensor operation and working mechanism, a schematic pressure-sensing model was created as illustrated in Fig. 1c–e. When pressure is applied to the sensor, the BP@PANI-coated fabric layers come closer to each other and generate conductive paths, resulting in a drop in resistance of the fabric and a rapid increase current (Fig. 1d). On the other hand, after releasing pressure on the sensor, the curvature of the fabric recovers and the contact area between fabrics layers is minimized (Fig. 1c). The total resistance of the sensor (RS_Total_) comprises two parts: the resistance of BP@PANI-coated fabric (RS_Fabric_) and the interface resistance between the copper tap and BP@PANI-coated fabric (RS_Interface_), which is defined as (RS_Total_) = (RS_Fabric_) + (RS_Interface_).

**Fig. 2:**
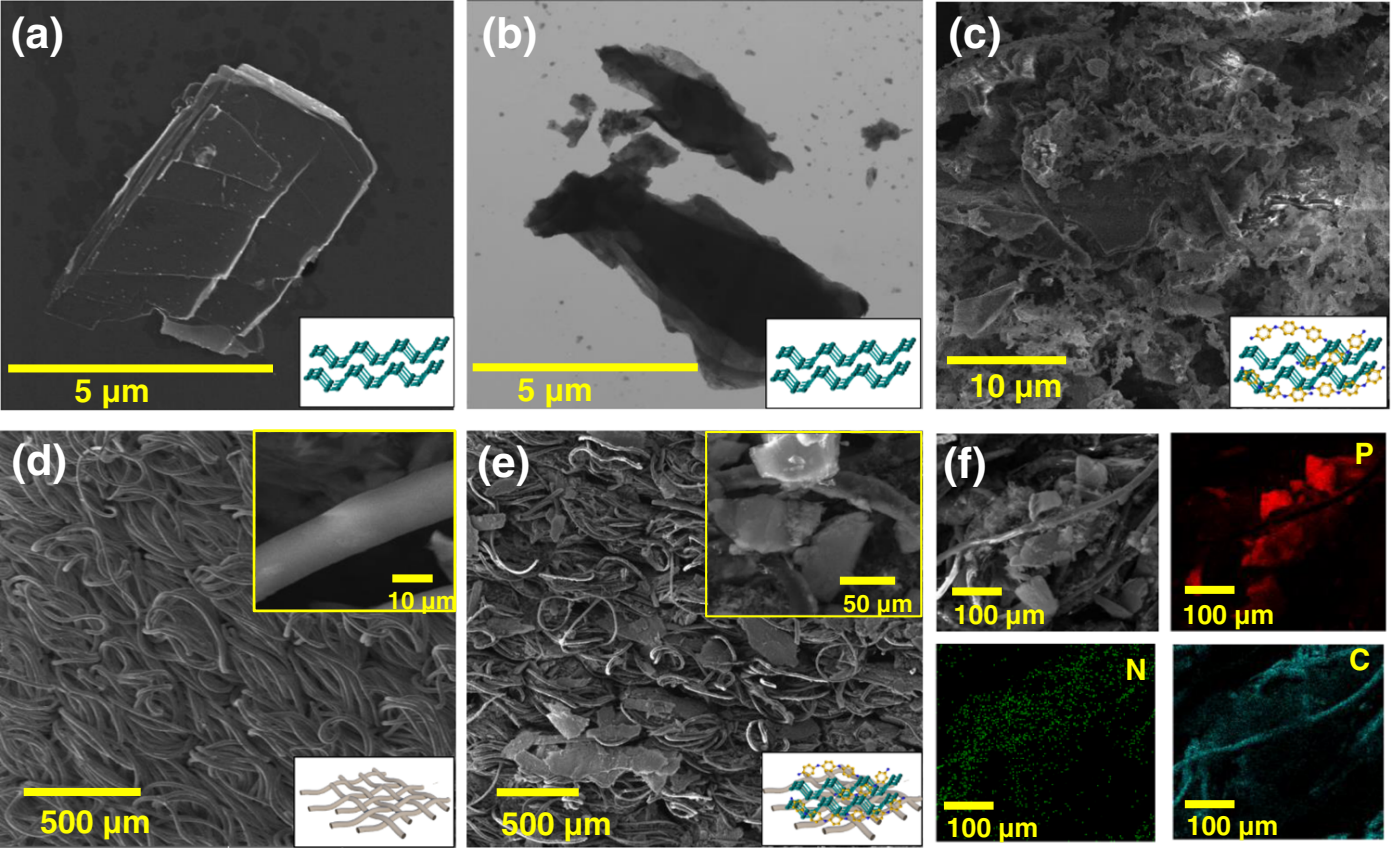
Morphology characterizations. **a**, **b** SEM and STEM images of pristine BP. **c** SEM image of BP@PANI composite. **d** SEM image of the pristine fabric. **e** SEM image of BP@PANI-coated fabric. **f** EDS mapping of BP@PANI-coated fabric.

Based on this sensing approach, the assistive auditory feedback application of the BP@PANI sensor is discussed in the following section. However, it is important to understand first the sensor’s structural and electrochemical properties. The surface morphology of the pristine BP and BP@PANI on fabric was observed by scanning electron microscopy (SEM), scanning transmission electron microscopy (STEM), and energy-dispersive spectroscopy (EDS). The SEM and STEM images in Fig. 2a, b clearly show the lamellar morphology of pristine BP. After in situ polymerizations of the aniline monomer, a sponge-like nanostructure of polyaniline (PANI) on BP surface was observed, see Fig. 2c. This sponge-like nanostructure may benefit for the fast response of tactile sensor. The SEM images of bare fabric with randomly oriented are represented in Fig. 2d. Such a structure enables a greater air gap between the fibers. Also, the bare fabric had circular finest fibers which may provide a vast surface area for the adhesion of BP@PANI composite. In Fig. 2e, an SEM image of BP@PANI-coated fabric shows that the fabric surface is fully wrapped with a BP@PANI composite. The cluster network and densely packed BP@PANI composite on fabric further improved the surface roughness and electrical properties. EDS mapping was carried out to verify the uniformity of BP@PANI composite and element distribution on the fabric. Figure 2f shows an even spread of P, N, and C elements, implying that BP@PANI is distributed uniformly throughout the fabric network.

Fourier-transform infrared (FTIR) spectroscopy was used to confirm the chemical structure of BP@PANI. FTIR spectra of pristine BP exhibited a stretching vibration of the P–O, P = O, and –OH bonds at 1058, 1328, and 2523 cm^−1^, respectively (Supplementary Fig. 3a)^24,35^. For the PANI, the peak around 823 and 879 cm^−1^ is responsible for the C–H stretching of the para-conjugated benzene ring. The bending and stretching (C–C) vibrations of the benzenoid and quinoid rings are attributed to the peaks at 1189, 1500, and 1619 cm^−1^, respectively^36^. The peaks that appeared at 1249 and 2487 cm^−1^ correspond to the C–N tertiary aromatic stretching mode and N–H stretching vibrations, respectively. The FTIR spectra of the BP@PANI composite revealed all the major peaks, indicating that both PANI and BP are present in the composite. Further, X-ray diffraction (XRD) measurements were performed to confirm the crystalline structure of BP after the polymerization of PANI (Supplementary Fig. 3b). The crystallinity of the BP is revealed by the occurrence of peaks in the (020), (040), (060), and (041) planes, which is comparable with prior findings^37,38^. Raman analysis of the BP@PANI composite (Supplementary Figure 3c) revealed that the intensity of BP peaks (360, 436, and 464 cm^−1^)^24,39^ reduced after the insertion of PANI and formed primary distinctive peaks of PANI at 1134 and 1167 cm^−1^ (C–H stretching vibration of aromatic ring), and 1515 and 1600 cm^−1^ (C–N and C–C stretching of the quinoid and benzenoid ring, respectively)^24^.

### Electrochemical properties of the BP@PANI-based tactile sensor

**Fig. 3:**
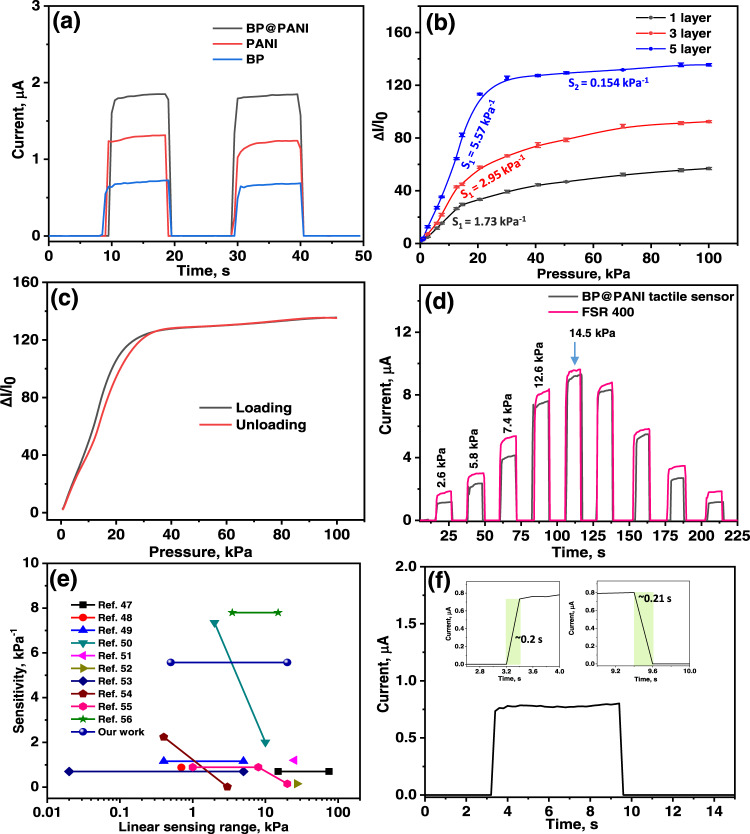
Electrochemical performance of BP@PANI-based tactile sensor. **a** Current response of pristine BP, PANI, and BP@PANI composite (tactile sensor with three layers samples coated fabrics and applied pressure of 5.8 kPa). **b** Pressure–response sensitivities curves for tactile sensors fabricated with a different layer of BP@PANI-coated fabrics (The error bars in relative current response of tactile sensor represent standard deviation based on three measurements). **c** Hysteresis loops of the tactile sensor at different applied pressure levels. **d** Response curve of the BP@PANI based tactile sensor and commercial (FSR400) sensor upon dynamic loading and unloading at different pressure levels. **e** Comparison of sensitivity between our study and the previously published pressure sensors. **f** Response/recovery of BP@PANI-based tactile sensor under a pressure of 1 kPa.

The electromechanical performance of BP@PANI-based tactile sensor has been examined and the results are depicted in Fig. 3. Sensitivity, response time, operating range, and stability are important factors to be considered while evaluating tactile sensor performance. The current output as a function of pressure was determined by applying a DC voltage (0.1 V) to the tactile sensor during all tests. The sensitivity of pristine BP, PANI, and BP@PANI composite as a function of pressure is shown in Fig. 3a, clearly illustrating that sensitivity of the BP@PANI composite is superior to that of pristine BP and PANI. In the case of pristine samples, the conductive path on the fabric may not be complete, resulting in the low response observed. These results show that PANI plays an important role in enhancing the conductivity path of BP for pressure sensing. In general, sensitivity is defined by *S* = (Δ*I*/*I*_0_)/ΔP^40,41^, where Δ*I* = (*I* − *I*_0_) and I_0_ and I indicate the current response before and after applied pressure, respectively. *P* denotes the amount of pressure exerted on the sensor. Figure 3b indicates that increasing the number of layers in the BP@PANI tactile sensor resulted in a significant gain in sensitivity in the low-pressure range. The explanation behind the variability in sensitivity (*S*) of sensors with varying active layers is because of the porous structure of the fabric and the presence of air spaces (curvature) in between layers. It was found that the five-layer tactile sensor has the greatest sensitivity of 5.57 kPa^−1^ with high linearity of *R*^2^ = 0.98 in the range of 0.5–20 kPa. This could be owing to the low modulus of the tactile sensor for the existence of significant air void layers and a porous network of BP@PANI-coated fabric. A small pressure stimulation can shrink the air space and improve the contact area between active layers, which provides high sensitivity. Then the sensitivity dropped to 0.154 kPa^−1^ in the pressure between 30 and 100 kPa. Above 20 kPa, the relative change in current appears to be nearly saturated making it difficult to determine the pressure quantitatively. The results show that the applied pressure raised further, the contact area of the BP@PANI coated fabrics and the distance between layers would be limited. Hence, five layers tactile sensor exhibits low sensitivity at high pressure. In comparison, the single- and three-layer tactile sensors had a sensitivity of 1.73 and 2.95 kPa^−1^, respectively, with a pressure range from 0.5 to 20 kPa. To clarify the obtained results, the sensing mechanism in terms of the tunneling effect between multilayers is detailed in Supplementary Note 1 and Supplementary Figure 4.

Figure 3c depicts the relative variation in current for the BP@PANI-based tactile sensor during the pressure loading/unloading cycle. Hysteresis is defined as ratio of the area beneath the ΔI/I_0_ vs pressure curves under loading and unloading. Hysteresis percentage was calculated using the following Eq. (1).

1 $${{{{{\rm{Hysteresis}}}}}}\,(\%)=\frac{{A}_{{{{{{\rm{Unloading}}}}}}}-{A}_{{{{{{\rm{Loading}}}}}}}}{{A}_{{{{{{\rm{Loading}}}}}}}}\times 100\%$$

where *A*_Loading_ and *A*_Unloading_ represent the area of the curves under loading and unloading, respectively. The calculated hysteresis was 3.43%, which was lower than many existing tactile sensors^42–46^. Low hysteresis proved the good reliability of the BP@PANI-based tactile sensor, which is critical for real-world applications, particularly in dynamic pressure events.

Figure 3d depicts the I–T curves of the sensor at various applied pressures. We can observe that as the applied pressure increases so does the current responsiveness, implying that the sensor’s conductive route lengthens and total resistance reduces. The obtained results demonstrated that the BP@PANI sensor is able to distinguish between various amounts of external force. Notably, the sensing response of our sensor perfectly matches with the commercial available pressure sensor (FSR 400), see Fig. 3d. In terms of lower pressure detection range and hysteresis percentage of BP@PANI based tactile sensor outperformed the FSR400 (low-pressure detection = ~1 kPa and hysteresis percentage = +10%). In addition, the relationship between current and applied voltage investigated was from −0.5 to 0.5 V. Supplementary Figure 5 shows that the I–V curves of the BP@PANI sensor have outstanding linearity over a wide applied pressure range, revealing better ohmic contact for the BP@PANI-coated fabric and copper electrode. In comparison with existing sensors (Fig. 3e), the five-layer BP@PANI-based tactile sensor shows excellent liner response to a wide range of pressure. There are many sensors based on carbon family (i.e. graphene and carbon nanotube), conducting polymers (i.e. polyaniline, polypyrrole and poly(3,4-ethylene dioxythiophene) polystyrene sulfonate) and their composites have liner ranges of less than 10 kPa^47–56^. However, very few sensors had a linear range greater than 10 kPa, although sensitivities were quite low. Although some sensors showed a higher sensitivity than ours, they had a nonlinear response or could be accomplished at low pressures.

**Fig. 4:**
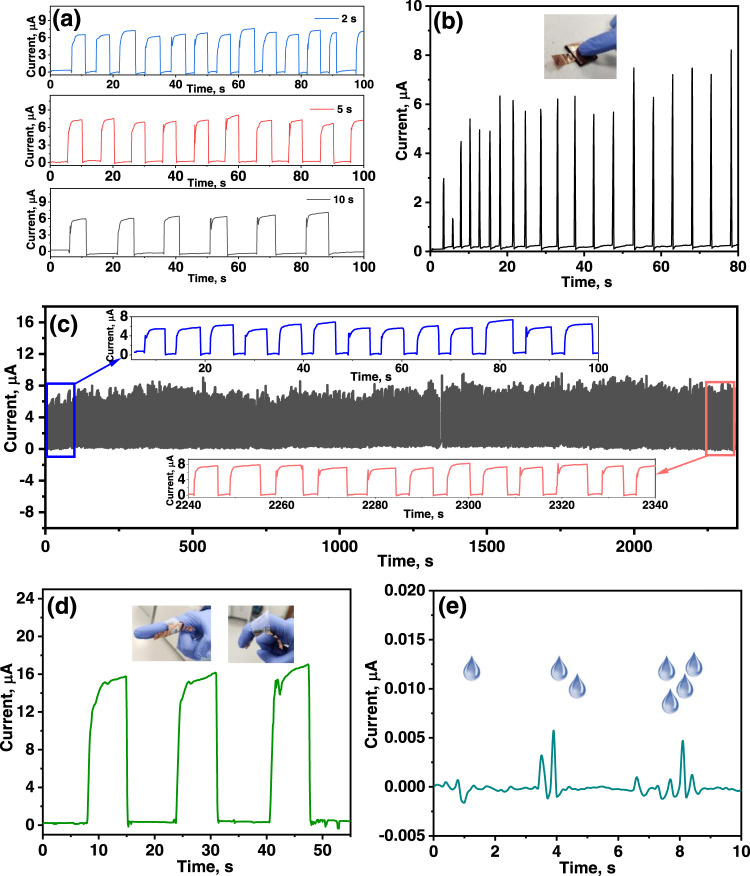
Real-world application. **a** Response curve with different speed applied pressure of 12 kPa. **b** Current response caused by finger touching on a sensor. **c** Cycling stability of BP@PANI-based tactile sensor upon applied pressure of 12 kPa (the inset shows a close-up of the first and last few cycles). **d** Current response upon finger movement (inset: digital image of a sensor attached to a finger in bend and unbend positions). **e** Sensor response detecting varying numbers of water drops.

In order to obtain a good response and recovery times of the BP@PANI sensor, we chose applied pressure of 1 kPa (Fig. 3f). Due to the tactile sensor has good sensitivity at low pressure, as illustrated in Fig. 3b. Under pressure, the response and recovery time of the BP@PANI sensor are 0.20 and 0.21 s, respectively, showing that the BP@PANI-coated textile structure in the sensor offers reasonable response properties. Besides, Fig. 4a exhibits remarkable uniformity and stability at various speeds of the applied pressure. To further, examine the long-term mechanical stability of the BP@PANI sensor, 350 cycles of loading/unloading pressure were measured. According to Fig. 4c, the current response exhibits some distortion during cyclic testing but maintains the same current difference after each load/unload pressure cycle, which indicates the sensor has good stability. Taking advantage of outstanding sensing capabilities, such as high sensitivity and reasonable response and recovery times, the BP@PANI-based tactile sensor might be utilized as an auditory human-machine interface.

### Real applications of the BP@PANI-based tactile sensor

The as-prepared BP@PANI-based tactile sensor is thin, lightweight, and robust in nature thanks to the fabric substrate, which allows it to be easily attached to various regions of the human skin or body. As shown in Fig. 4b, human finger tapping can be detected by intense current peaks due to the high sensitivity and reasonable response/recovery time of the BP@PANI-based pressure sensor. It was observed that the peaks may fluctuate in intensity during repetitive tapping tests, which may be produced by unevenly applied pressure to the surface of sensor. Other finger actions, such as straightening and bending circularly, are accurately recorded as current signals with the sensor attached to the finger joint (Fig. 4d). When the finger is straight, no pressure is applied to the associated sensor and the relative current remains unchanged. Once the user bends their finger, pressure is delivered to the sensor, leading to the resistance drop visible in real-time I-T curves. The precise on/off current changes demonstrate excellent sensitivity and reasonable response while the relatively consistent peak intensities in each curve verify the sensor’s stability in operation time. Further, pay attention to the low-pressure detection limit, when a single drop of water drips on the sensor, a very small and fast noticeable change would be observed in current signals as shown in Fig. 4e. In addition, the sensor can distinguish responses toward low-pressure changes generated by varying numbers of drops. Supplementary Figure 6 shows the tactile sensor attached to the human neck (carotid artery) for real-time monitoring of the physical force induced by the heartbeats, showing consistent pulse patterns with around 78 beats per minute. These outcomes provided a good example for HMI and health monitoring applications.

**Fig. 5:**
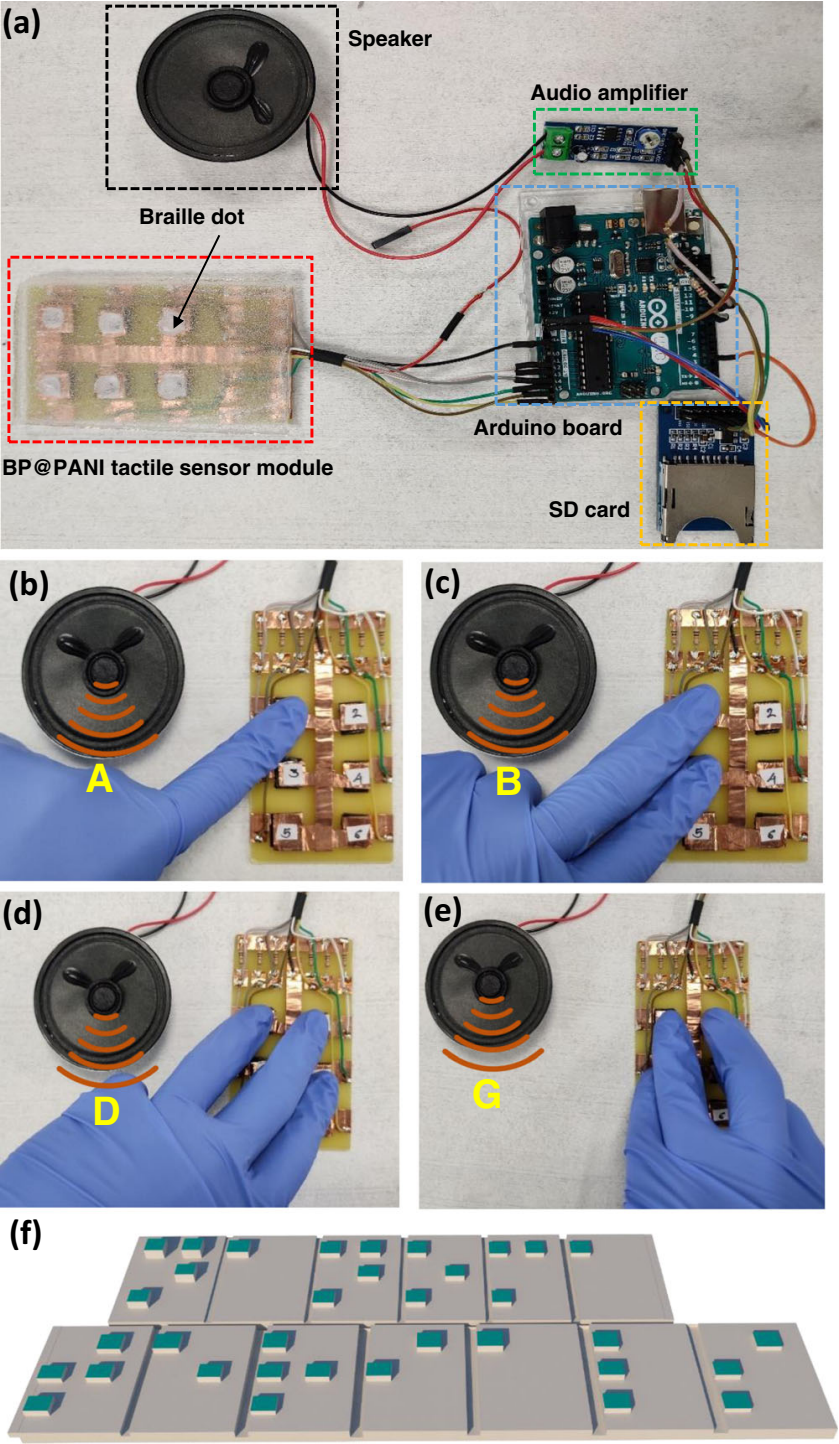
BP@PANI-based tactile sensor for auditory feedback. **a** Digital photograph of six-pixel BP@PANI-based tactile sensor array for press-to-audio feedback. **b**-**e** Digital photographs of diverse braille alphabet patterns converted to audio signals. **f** Press-to-audio illustrates converting braille pattern “nanomaterials” to audio signals.

To investigate the potential application as an auditory human-machine interface, we fabricated a six-pixel tactile sensor array that meets the typical braille character set to read alphabet letters corresponding to pressed sensors. A digital photograph of the connection is shown in Fig. 5a and Supplementary Figure 7. Its microcontroller unit (MCU) and analog-to-digital converter in the circuit were employed to acquire and digitize the voltage signals from the pressure sensor array and convert them into audio through an audio amplifier (Supplementary Note 2). Each of the six-pressure sensors has a different voltage output in both the original and pressed states (Supplementary Table 1). The system was coded to receive voltage readings from the six-sensor array and play the relevant word audio clip based on which sensors were pressed and which were not (Supplementary Table 2). Figure 5b–e depict the press-to-audio transition for different braille letters, such as A, B, D, and G, which correspond to one, two, three, and four sensors pressed, respectively (Supplementary Movie 1). In addition, we were able to correctly transform the word “nanomaterials” into audio (Fig. 5f and Supplementary Movie 2). Most importantly, we successfully demonstrate press-to-audio dialog between humans and machines, by converting five braille words to audio signals such as “hello, good, no, yes and ok” (Supplementary Movie 3). Diverse field applications of the BP@PANI tactile sensor offer a superior possibility to build assistive communication devices that can improve the quality of life for people with communication disabilities.

## Discussion

Here, we demonstrated a prototype auditory feedback device for a human-machine interface. By sandwiching BP@PANI-coated fabric between two conducting electrodes, the fabricated piezoresistive tactile sensor exhibited excellent sensitivity, steady cycling stability, good pressure range, and reasonable response time. An integrated six-pixel BP@PANI-based tactile sensor was used to convert the pressured text in diverse braille patterns into audio and correctly pronounced alphabets. The proposed touch-to-audio approach may be very useful for people with communication disabilities to learn and read braille letters as well as improve their communication ability. It can also be used to create portable electronic books. Remarkably, the whole device fabrication process is scalable and does not necessitate the use of complex and expensive machinery. To the best of our knowledge, the BP have never been reported before in wearable tactile sensor and auditory feedback system. We believe that our approach will pave the way for low-cost tactile sensors that can be easily integrated into future wearable electronics such as human-machine communication interfacing and touch screens.

## Methods

### Materials

Black phosphorous, aniline, dimethylformamide, ammonium persulfate, and hydrochloric acid were purchased from Sigma-Aldrich. Dimethylsiloxane (PDMS) was purchased from Biesterfeld AG, Germany. For the design circuit, various electrical components were purchased from the local electrical shop in the Czech Republic. Polyester/cellulose-based fabric was purchased from VWR International, LLC.

### Preparation of BP@PANI-coated fabric

A highly dispersed BP solution at a concentration of 0.36 mg/mL was prepared using an ultrasonic treatment for 2 h. A piece of polyester/cellulose blend fabric was immersed into the BP dispersion for 30 s, then taken from the BP solution and dried in the oven at 55 °C for 3 h. Afterwards, 100 mM aniline was added to 10 mL of 1 M HCl solution and stored in an ice-water bath. In the same manner, APS was poured into 5 mL of 1 M HCl and kept in an ice-water bath. Next, the BP-coated fabric was soaked in the aniline monomer solution for 30 min and then APS solution was added. The fabric was removed after 24 h and cleaned with deionized water, and the BP@PANI fabric was realized.

### Preparation of BP@PANI tactile sensor and tactile sensor array

The multilayer BP@PANI-based tactile sensor is made up of five layers of BP@PANI fabric. The size of each fabric is 1.5 cm × 1.5 cm. Copper electrodes were applied to the top and bottom layers. The sensors were wrapped in a transparent plastic sheet to maintain conformal contact between the BP@PANI fabric and the electrode. To optimize device performance, the different layers (1, 3, and 5) of BP@PANI-based sensors were also fabricated. For the fabrication of wearable six pixels tactile sensor array, six BP@PANI based tactile sensors were mounted onto poly-dimethylsiloxane (PDMS) substrate with kept 1.5 cm spacing between tactile sensors. The braille dots were prepared by PDMS and placed on top of the six sensors. Supplementary Figure 2f demonstrates the schematic of a six-pixel tactile sensor array. To keep the tactile sensor straight while wearing or bending, a rigid substrate (acrylic lightweight plexiglass) was placed between the sensor and a flexible PDMS substrate.

### Characterization and measurements

The structure and morphology of pristine BP and BP@PANI composite were characterized by scanning electron microscopy (Maia 3, Tescan), energy-dispersive X-ray spectroscopy (EDS, SDD detector X-MaxN 80TS), X-ray powder diffraction (Bruker D8, Germany), attenuated total reflection Fourier-transform infrared spectroscopy (Thermo-Nicolet, PIKE Technologies, USA), and Raman analysis (Thermo Scientific DXR Raman Microscope). The electrochemical performance of the BP@PANI-based tactile sensor was tested on an electrochemical workstation (PGSTAT204 Autolab, Netherland) and data analyzer by Nova 1.1 software.

## Supplementary information

Supplementary Information

Description of Additional Supplementary Files

Video S1

Video S2

Video S3

## Data Availability

The data generated in this study are provided in the paper or its supplementary Information and FigShare repository (10.6084/m9.figshare.21311748).
