# Supplementary material for: Black phosphorous-based human-machine communication interface
Source: Nat Commun. 2023 Jan 3;14:2. doi: 10.1038/s41467-022-34482-4 (PMC9810665; doi:10.1038/s41467-022-34482-4)
Supplement: Supplementary file 1 — Supplementary Information [file 41467_2022_34482_MOESM1_ESM.pdf]

Supplementary Information

Black Phosphorous-based Human-Machine Communication Interface

Jayraj V. Vaghasiya,[1] Carmen C. Mayorga-Martinez,[1] Jan Vyskočil,[1] Martin Pumera[1,2,3,4]*

[1]Center for Advanced Functional Nanorobots, Department of Inorganic Chemistry, Faculty of Chemical Technology, University of Chemistry and Technology Prague, Technická 5, 166 28 Prague, Czech Republic

[2]Department of Chemical and Biomolecular Engineering, Yonsei University, 50 Yonsei-ro, Seodaemun-Gu, Seoul 03722, Korea

[3]Faculty of Electrical Engineering and Computer Science, VSB - Technical University of Ostrava, 17. listopadu 2172/15, 70800 Ostrava, Czech Republic

[4]Department of Medical Research, China Medical University Hospital, China Medical University, No. 91 Hsueh-Shih Road, Taichung 40402, Taiwan

E-mail: pumera.research@gmail.com

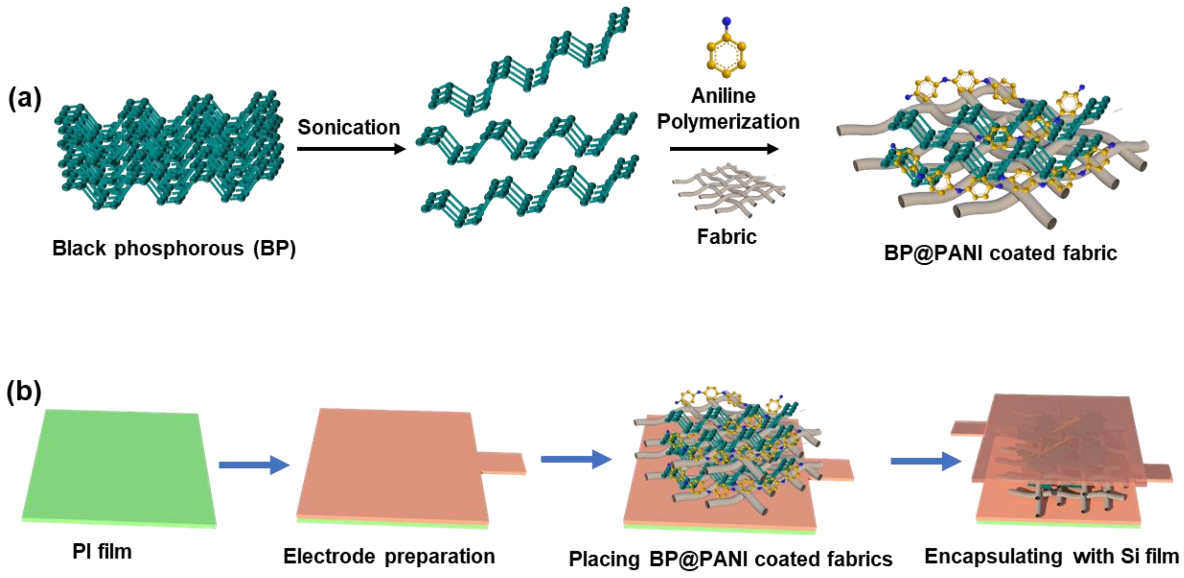

Supplementary Figure 1. Schematic fabrication of the BP@PANI based piezoresistive tactile sensor. (**a**) The preparation procedure of BP@PANI based fabric, and (**b**) Fabrication process of BP@PANI based tactile sensor.

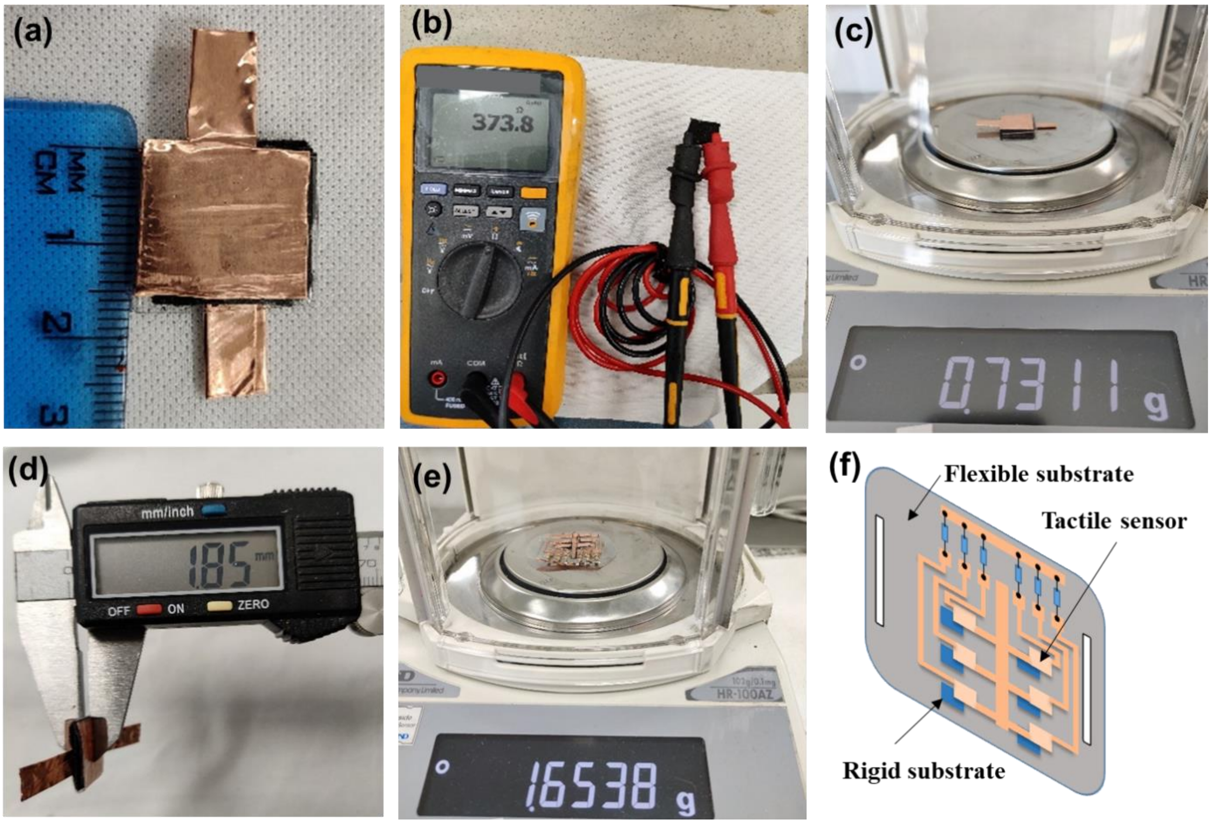

Supplementary Figure 2. Structural geometry of BP@PANI based tactile sensor**. (a)** Photographs of BP@PANI based tactile sensor with scale bar, **(b)** resistivity of single layer BP@PANI coated fabric, **(c)** weight of BP@PANI based tactile sensor, **(d)** thickness of BP@PANI based tactile sensor, **(e)** weight of six-pixel tactile sensor array and **(f)** schematic view of a six-pixel tactile sensor array, where flexible and rigid substrates made from polydimethylsiloxane and acrylic lightweight plexiglass, respectively.

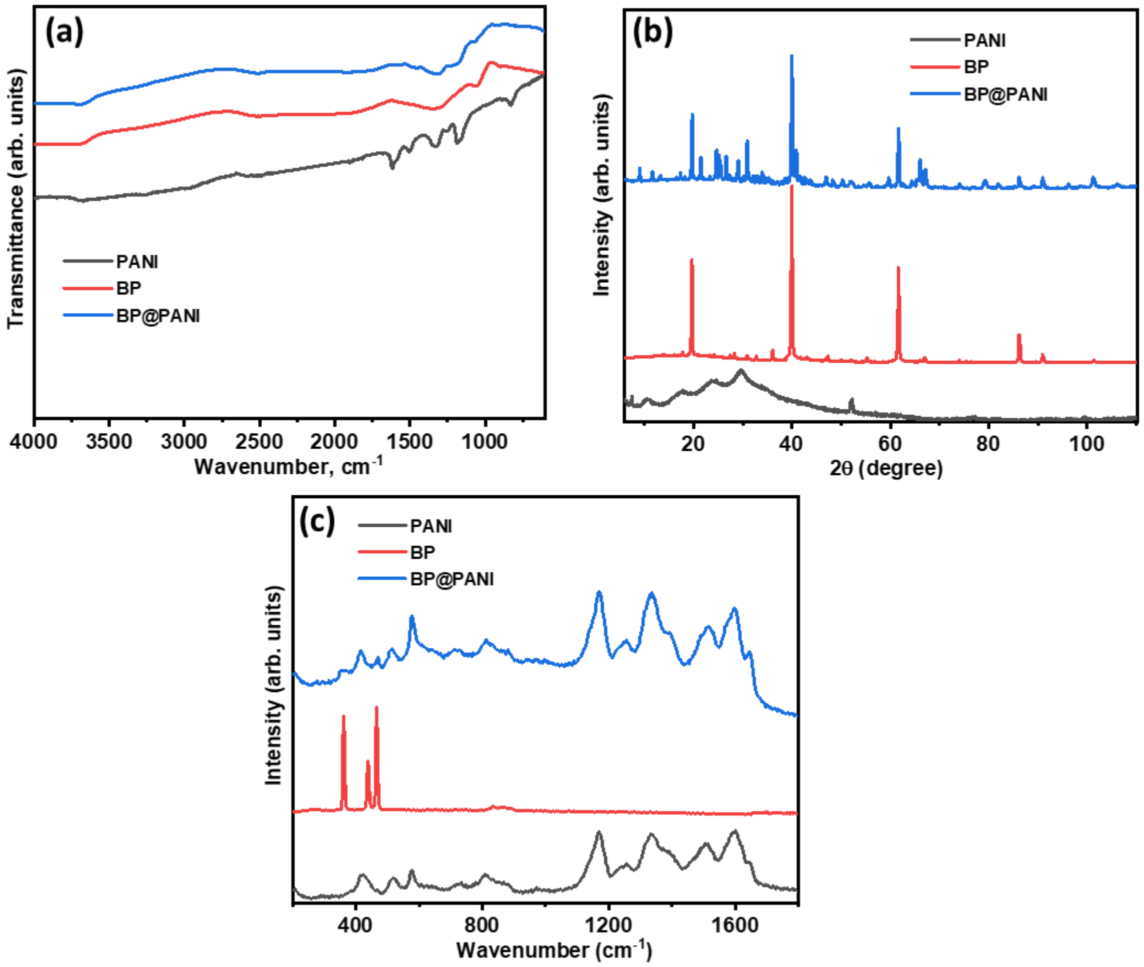

Supplementary Figure 3. Structural characterization of pristine BP, PANI and BP@PANI composite. (**a**) ATR-FTIR spectra (Note: The ATR-FTIR spectra of samples correspond well to the reflection spectra of conducting samples, in which the interaction with free charge-carrieres in the protonated state of PANI or BP is reflected. Baseline correction disturb the spectra and finally change them into the spectra of non-conducting deprotonated samples.[1,2] However, a broad absorption band typical of the conducting form of PANI can be observed at wavenumbers higher than 2000 cm^{-1}.[3]), (**b**) XRD pattern, and (**c**) Raman spectroscopy.

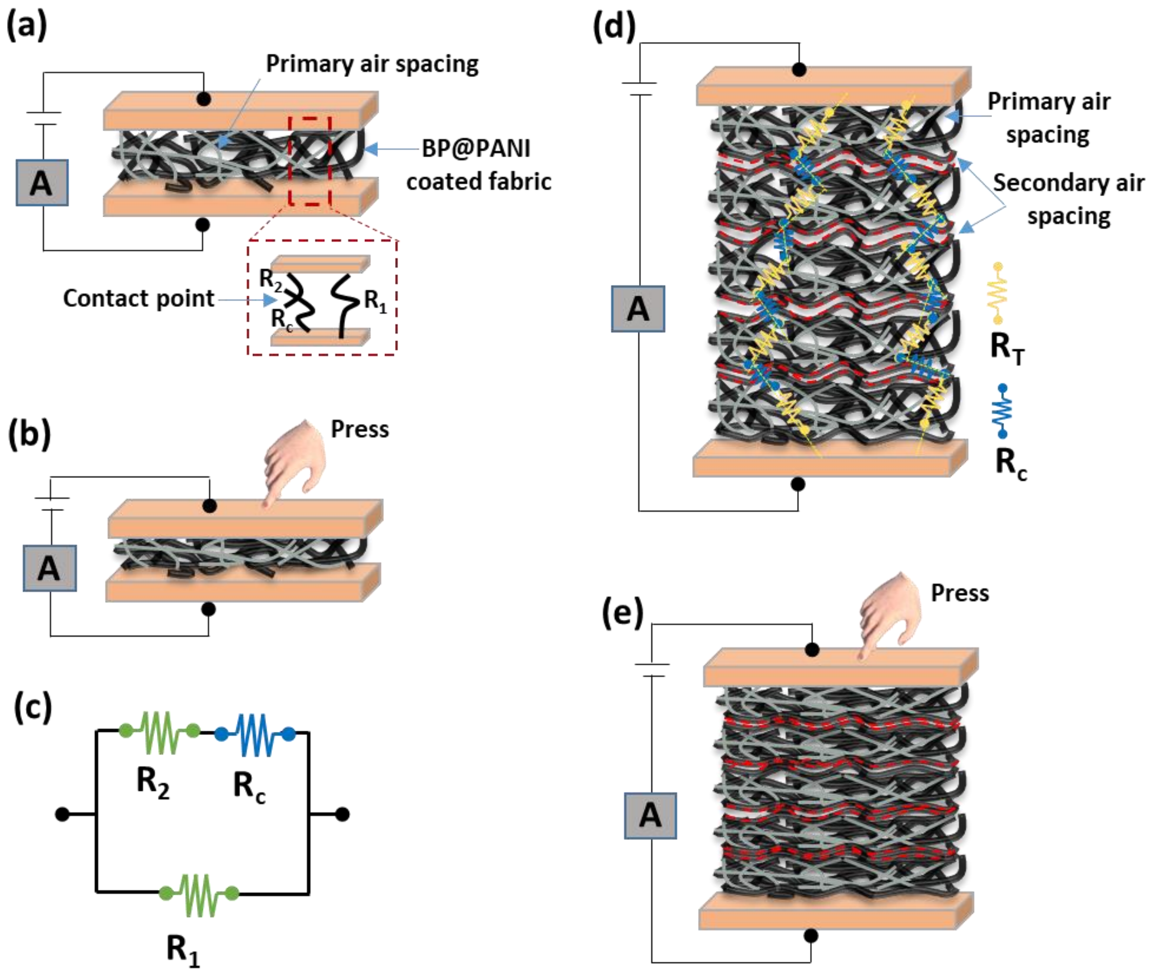

Supplementary Figure 4. Schematic models for the sensing mechanism of single and five-layer BP@PANI based tactile sensors. **(a, b)** circuit diagram of single layer BP@PANI based tactile sensor without and with applied pressure, **(c)** the resistance model of single layer BP@PANI based tactile sensor, **(d, e)** circuit diagram of five-layer BP@PANI based tactile sensor without and with applied pressure.

Supplementary Note 1. Sensing mechanism of single and five-layer BP@PANI based tactile sensors.

To better comprehend the operation of single and multilayer BP@PANI-based tactile sensors, we developed simple schematic models as shown in Supplementary Figure 4. The circuit model of the single-layer tactile sensor is shown in Figures 4a and b, where total resistance (R_T) is calculated from the resistance of each fiber of fabric (R_1) and the contact resistance between neighboring fibers (R_c) (Figure 4c). As a result, the entire resistance might be described as Eq. (1).

$$R_T = 1 \Big/ \frac{1}{R_1} + \frac{1}{2R_1 + R_c} = 1 \Big/ \frac{1}{R_1} + \frac{1}{R_2 + R_c} \quad \ldots\ldots\ldots\ldots \quad (1)$$

Holm's theory (Eq. (2)) states that the R_c is attributed to the resistivity of the fibre *(ρ),* fiber contact hardness (H), the contact pressure (P), and the number of contact sites (n).[4]

$$R_C = \frac{\rho}{2}\sqrt{\pi H / nP} \quad \ldots\ldots\ldots\ldots\ldots\ldots \quad (2)$$

When a single layer tactile sensor was subjected to small pressure, the number of contact sites enhanced due to the removal of the air void between the fibers of the fabric, resulting decrease the resistance.[5] In general, R_c is substantially greater than R_1, implying that R_1 plays a key role in overall resistance. Due to the high conductivity (R_s = 373 **Ω**) of the BP@PANI coated fabric, the tactile sensor has very less impact on the reduction of R_1. Therefore, the single-layer tactile sensor exhibits lower sensitivity.

In contrast, aside from the noticeable increase of primary air void among the BP@PANI coated fabrics, the five-layer (or multilayer) tactile sensor also has a secondary air gap between the neighbouring fabric with rough surfaces (Figures 4d and e). Hence, even at low pressure, the

gap in multilayer tactile sensors dropped dramatically and many electrical paths were created, resulting in a substantial drop of resistance.

The total resistance of the multilayer tactile sensor is determined by the fabric bulk resistance (R_T) and contact resistance between neighboring layers (R_c), which are interconnected in series configuration (Eq. (3)). Compared to R_c, the R_T can be eliminated (Eq. (4)). According to Holm's theory, can be determined as given in Eq. (5).

$$R_0 = 4R_C + 5R_T \quad \ldots\ldots\ldots\ldots.. \quad (3)$$

$$R_0 = 4R_C \quad \ldots\ldots\ldots... \quad (4)$$

$$R_0 = 4R_C = 4 \times \frac{\rho}{2}\sqrt{\pi H / nP} \quad \ldots\ldots\ldots\ldots.. \quad (5)$$

When applied pressure (P) is raised, n rapidly and dramatically increases, resulting in a sharp drop of contact resistance and obtaining high sensitivity of BP@PANI-based tactile sensor. The contact area of the BP@PANI coated fabrics and the distance between layers would be limited as the applied pressure further increased. Therefore, the multilayer tactile sensor has lower sensitivity at high pressure and high sensitivity at low pressure (0.5 to 20 kPa). To conclude, the air space between active layers and circuit connection mode both play essential roles in increasing tactile sensor sensitivity and the disparity in sensitivity between single and five-layer tactile sensors.

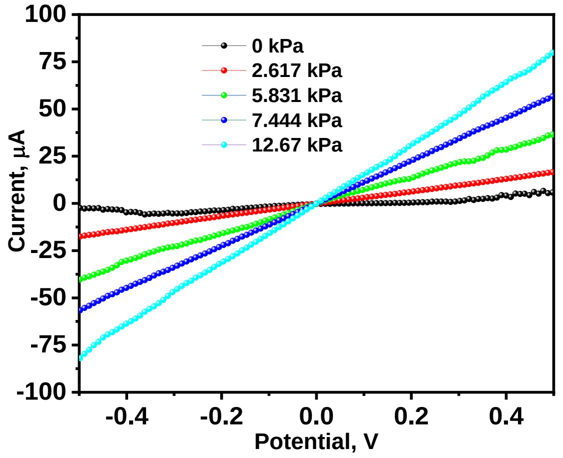

Supplementary Figure 5. Electrochemical study. I-V curves of BP@PANI-based sensor with different applied pressure.

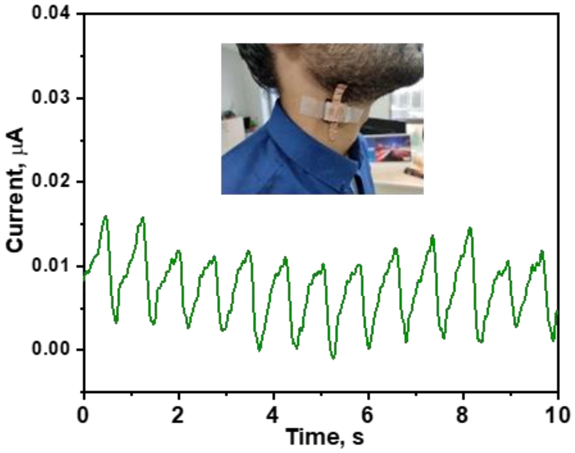

Supplementary Figure 6. Health monitoring application. The current response of the tactile sensor mounted on the neck for monitoring carotid artery pulse waves.

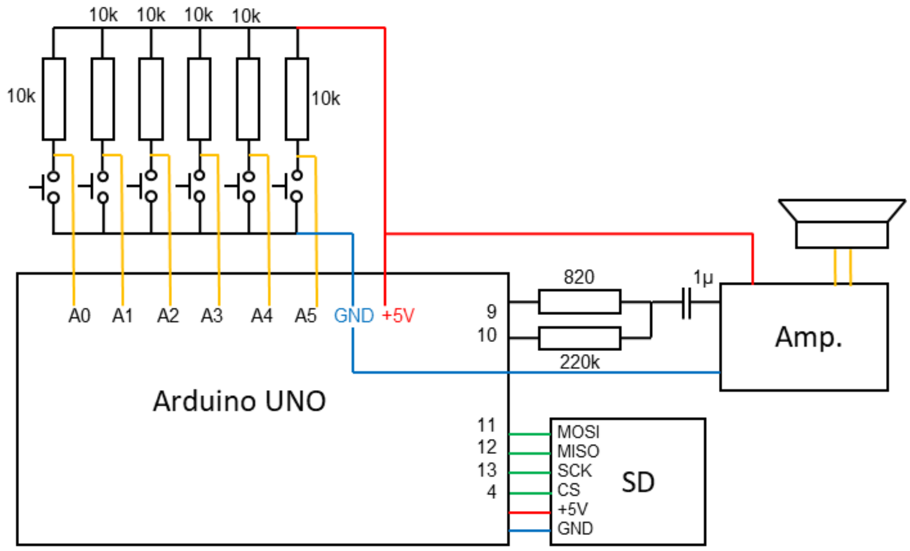

Supplementary Figure 7. Electronic interface**.** Circuit diagram of BP@PANI tactile sensor based braille keyboard.

Supplementary Note 2. Press to audio Interface Design

The Braille keyboard uses six BP@PANI pressure sensors. Each of them is connected across 10 k Ohm resistor to +5 V. The voltage of each sensor is measured by 10 bit AD converter of Arduino UNO. Due to variations in pressure sensors´ characteristics, six different decision levels were selected for the respective sensors. A voltage difference was discovered between the pressed and un-pressed states of sensors (Supplementry Table 1). Six voltages are measured simultaneously and assigned to a six-logical voltage percentage. When a specific combination of pixels in the pressure sensor was pressed, the voltage reading from all the individual pixels was acquired by the microcontroller. If the voltage percentage falls below a predetermined level, the signal is passed through a microcontroller, which activates the speaker and plays an audio pronunciation of the English letter. The corresponding letter is then selected from 26 lines

of look-up Supplementary Table 2. The audio files of letters are stored on an SD card. This card is connected to the Arduino board. Audio processing uses the simple SDAudio library, https://hackerspace-ffm.de/wiki/index.php?title=SimpleSDAudio. The whole device is configured as 16-bit mono output and uses two PWM outputs. The resulting signal is amplified with an audio amplifier based on the module with LM386 integrated circuit and connected to a speaker.

Supplementary Table 1. Voltage response upon the pressed and un-pressed state.

**Tactile sensor**	**1**	**2**	**3**	**4**	**5**	**6**
**Un-pressed (H)**	2.0 V	4.8 V	1.35 V	2.26 V	2.98 V	3.7 V
**Pressed (L)**	0.5 V	0.5 V	0.3 V	0.5 V	0.78 V	0.25 V

Supplementary Table 2. Six-pixel sensor braille pattern corresponding English letter**.**

1	2	3	4	5	6	Alphabets
L	H	H	H	H	H	A
L	H	L	H	H	H	B
L	L	H	H	H	H	C
L	L	H	L	H	H	D
L	H	H	L	H	H	E
L	L	L	H	H	H	F
L	L	L	L	H	H	G
L	H	L	L	H	H	H
H	L	L	H	H	H	I
H	L	L	L	H	H	J
L	H	H	H	L	H	K
L	H	L	H	L	H	L
L	L	H	H	L	H	M
L	L	H	L	L	H	N
L	H	H	L	L	H	O
L	L	L	H	L	H	P
L	L	L	L	L	H	Q
L	H	L	L	L	H	R
H	L	L	H	L	H	S
H	L	L	L	L	H	T
L	H	H	H	L	L	U
L	H	L	H	L	L	V
H	L	L	L	H	L	W
L	L	H	H	L	L	X
L	L	H	L	L	L	Y
L	H	H	L	L	L	Z

Supplementary References

1. Stejskal, J. et al. Conducting polymers: polyaniline, *Encycl. Polym. Sci. Technol.*, Wiley Online Library: Hoboken, NJ, USA, pp. 1-44 (2015).

2. Trchova, M., Moravkova, Z., Sedenkova, I. & Stejskal J. Spectroscopy of thin polyaniline films deposited during chemical oxidation of aniline, *Chem. Pap.* **66,** 415-445 (2012).

3. Epstein, A. J. et al. Insulator-to-metal transition in polyaniline: Effect of protonation in emeraldine. *Synth. Met.,* **21,** 63-70 (1987).

4. Li,Y. et al. Electronic textile by dyeing method for multiresolution physical kineses monitoring, *Adv. Electron.Mater.* **3,** 1700253 (2017).

5. Zhang, L. et al. Carbonized cotton fabric-based multilayer piezoresistive pressure sensors. *Cellulose* **26**, 5001-5014 (2019).
