# Supplementary material for: Black phosphorous-based human-machine communication interface
Source: Nat Commun. 2023 Jan 3;14:2. doi: 10.1038/s41467-022-34482-4 (PMC9810665; doi:10.1038/s41467-022-34482-4)
Supplement: Supplementary file 2 — Description of Additional Supplementary Files [file 41467_2022_34482_MOESM2_ESM.docx]

**Description of Additional Supplementary Files**

**File Name: Supplementary Movie 1
Description:** Show press-toaudio conversion for different braille letters from A to G.

**File Name: Supplementary Movie 2
Description:** Transform the word nanomaterials into audio.

**File Name: Supplementary Movie 3
Description:** Shows typical dialogue interaction between human and machine.
